# VvMYB15 and VvWRKY40 Positively Co-regulated Anthocyanin Biosynthesis in Grape Berries in Response to Root Restriction

**DOI:** 10.3389/fpls.2021.789002

**Published:** 2021-12-09

**Authors:** Dongmei Li, Zhenping Wang, Sijie Sun, Kun Xiao, Minghao Cao, Xiangyi Li, Chao Ma, Caixi Zhang, Lei Wang, Hongli Lian, Shiping Wang

**Affiliations:** ^1^Department of Plant Science, School of Agriculture and Biology, Shanghai Jiao Tong University, Shanghai, China; ^2^School of Agriculture and Biology, Ningxia University, Yinchuan, China; ^3^Institute of Agro-Food Science and Technology, Key Laboratory of Agro-Products Processing Technology of Shandong, Shandong Academy of Agricultural Sciences, Jinan, China

**Keywords:** anthocyanin, VvMYB15, VvWRKY40, root restriction, grape

## Abstract

In most grapevine planting regions, especially in south of China, plenty of rainfall and high water level underground are the characteristic of the area, a series of problem during fruit ripening easily caused poor color quality. Thereby affecting fruit quality, yield and economic benefits. The accumulation of anthocyanin is regulated by transcriptional regulatory factor and a series of cultivation measures, root restriction can make plants in the environment of stress and stress relief, root restriction induced the higher expression of *VvMYB15* and *VvWRKY40*, and consistent with anthocyanin accumulation. Whether and how root restriction-inducible VvMYB15 and VvWRKY40 transcription factor regulate anthocyanin synthesis in grape berry is still unclear. In this study, we identified that the transient overexpression of *VvMYB15* and *VvWRKY40* alone or both in strawberry fruits and grape berries can promote anthocyanin accumulation and increase the expression level of anthocyanin biosynthetic genes, indicating VvMYB15 and VvWRKY40 play a positive regulator of anthocyanin biosynthesis. Furthermore, we confirmed that both VvMYB15 and VvWRKY40 specifically bind to the promoter region of *VvF3*′*5*′*H* and *VvUFGT*, and the expression of *VvF3*′*5*′*H* and *VvUFGT* is further activated through the heterodimer formation between VvMYB15 and VvWRKY40. Finally, we confirmed that VvMYB15 promoted anthocyanin accumulation by interacting with VvWRKY40 in grape berries, our findings provide insights into a mechanism involving the synergistic regulation of root restriction-dependent coloration and biosynthesis *via* a VvMYB15 and VvWRKY40 alone or both in grape berries.

## Introduction

Color is an important fruit quality index that influence the purchasing behavior of consumers to some degree. As a type of secondary metabolite, anthocyanin constitutes one of main pigments responsible for fruit color. Additionally, health benefits of anthocyanin have been widely described, especially in the prevention of disease associated with oxidative damage, such as cardiovascular and neurodegenerative disease (Mattioli et al., 2020), protecting the liver from damage.

To date, multiple transcription factors (TFs) belonging to MYB, WD40, bHLH, and MADS have been reported to regulate anthocyanin biosynthesis. Among these TFs, MYB TFs belong to one of the largest plant TFs, some members have been reported to be involved in anthocyanin biosynthesis in horticultural crops, such as apple MdMYB10, highly ortholog of anthocyanin regulatory, such as Arabidopsis *PAP1* (Espley et al., 2007), MdMYB1, regulator of light-induced anthocyanin biosynthesis (Li et al., 2012) and MdMYB110a, ABA-responsive genes (Umemura et al., 2013), strawberry FaMYB10, bayberry MrMYB1, pear PyMYB10 and PyMYB114, transcriptional activators of anthocyanin structural genes (Yao et al., 2017) by forming the MBW transcriptional complex. MYBA1 and MYBA2 at the color locus are the major genetic determinants of grape skin color, and the mutation of two functional genes (VvMYBA1, VvMYBA2) from these loci lead to white skin color (Jiu et al., 2021), while VvMYB86 can inhibit anthocyanin biosynthesis primarily in grape berry by downregulating the expression of two structural genes including *VvANS* and *VvUFGT* (Cheng et al., 2021). Transcriptome and metabolic data revealed that up-regulated expression of VvMYBA1, VvMYBA5, VvMYBA6 and VvMYBA7 are responsible for the specific anthocyanin patterns of Yan73 vegetative tissues, with VvMYB5A and VvMYB6A likely encoding the major regulators of anthocyanin biosynthesis in Yan73 vegetative tissues, simultaneously, VvMYBC2-L1 and VvMYBC2-L3, as repressor genes, are activated and may negatively regulated anthocyanin biosynthesis in vegetative tissues, therefore maintaining the balance of anthocyanin level (Xie et al., 2020). VvMYBPA1 did not activate the promoter of *VvUFGT*, which encodes the anthocyanin specific flavonoid-3-*O*-glucosyltransferase, while activated the expression of *VvLAR* and *VvANR* and further regulate the proanthocyanidin synthesis, indicating that tissue and temporal-specific expression of *VvANR* and *VvLAR* correlates with proanthocyanidins (PAs) accumulation prior to veraison in grapes (Bogs et al., 2007). Another grapevine TF, VvMYB5a, was recently shown to be expressed in grapes prior to veraison but ectopic expression in tobacco affected the metabolism of anthocyanins, flavonols, suggesting that it controls a number of different branches of phenylpropanoid pathway (Deluc et al., 2006).

In addition to MBW complex, other TFs, such as NACs, ERFs, and WRKY, were found to be involved in anthocyanin biosynthesis. These TFs can regulate many physiological processes by indirectly or directly binding to the promoter of target genes. Recently, several reports have shown that WRKY protein have obvious correlation with the regulation of anthocyanin biosynthesis. For example, GbWRKY1 in Gossypium barbadense was proved to have a positive correlation with anthocyanin accumulation (Xu et al., 2012). In a previous study, AtWRKY75 responded to low phosphate (Pi) stress by decreasing anthocyanin accumulation in seedlings (Devaiah et al., 2007). AtWRKY6 promotes senescence and pathogen defense-associated gene (PR1) activity, which is related to senescence and pathogen defense, and plant responds to biotic and abiotic stress by decreasing anthocyanin accumulation (Robatzek and Somssich, 2002). Recently, it was reported that the WRKY TFs PhPH3 in petunia correlates with change in the color of petals by playing a role in downstream of MBW complex, VvWRKY26, a homologous gene of PhPH3 in grape berry, induces the accumulation of flavonoids (Amato et al., 2017). In apple, MdWRKY40 is a key modulator in wounding-induced anthocyanin biosynthesis (Xu et al., 2018). WRKY family was related to anthocyanin biosynthesis in red-skinned pear (Yang et al., 2015). However, whether WRKYs are involved in anthocyanin biosynthesis by interacting with the TFs MYB or bHLH in red grapevine is still unclear.

Root restriction, making plant in the environment of stress and stress release, could promote the anthocyanin content in grape berries, and transcription factor responds to various stresses. Expression level of *MYBA* controlling the expression of the anthocyanin-specific gene UFGT was significantly higher in fruits under water stress throughout ripening (Castellarin et al., 2007). Which one of the transcription factors regulate anthocyanin accumulation under root restriction, numerous of differentially expressed genes related to anthocyanin biosynthesis were selected as candidates from previous transcriptome data (Leng et al., 2016, 2020). Our analysis of the transcriptome data revealed TFs, namely VvMYB15 and WRKY40, were significantly increased. However, little information is available regarding the function of MYB15 and WRKY40, apart from a few reports, which showed that ectopic expression of AtMYB15 improved drought and salt tolerance in Arabidopsis, VvMYB15 was involved in the transcriptional regulation of stilbene synthesis (Hoell et al., 2013) and basal immunity in grape (Luo et al., 2019), MYB15 have not yet been reported to promote anthocyanin accumulation, it is noteworthy that whether VvMYB15 can promote anthocyanin accumulation in grape berries under root restriction, therefore, VvMYB15 and VvWRKY40 were transformed into strawberry fruits and grape berries by a transient expression system. In addition, qRT-PCR and pH differential method revealed VvMYB15 alone or together with VvWRKY40 can activate the activity of anthocyanin biosynthesis structural genes in strawberry fruits or grape berries. Furthermore, yeast-one-hybrid and dual-luciferase reporter system assay demonstrated that VvMYB15 alone or together with VvWRKY40 can activate the expression of *VvF3*′*5*′*H* and *VvUFGT*. Furthermore, yeast-two-hybrid, bimolecular fluorescence complementary and Co-IP confirmed the interaction of VvMYB15 with VvWRKY40. Our research revealed a potential mechanism of regulating anthocyanin biosynthesis in colored grape berry, which will help elucidate the regulatory network to clarify anthocyanin accumulation in other species.

## Experimental Procedures

### Plant Materials and Growth Conditions

The grape cultivar “Muscat Hamburg” (*Vitis vinifera* L.) was planted in greenhouse at Shanghai Jiao Tong University, Shanghai, China. About 60 grapevines with uniform growth vigor were selected. The cultivation medium was a mix of sand, loam, and perlite (1:1:1), all vines were placed at spacing of 2 m × 0.7 m in north-south row direction. Berries were sampled at 10-day intervals from 20 days after anthesis to 110 days after anthesis during 2019 growing season. Berries used for molecular biology experiments were sampled and immediately frozen in liquid nitrogen before storing at −80°C.

The strawberry plants (*Fragaria* × *ananassa* cv. “Hongyan”) were planted in greenhouse at Shanghai Jiao Tong University, Shanghai, China.

Tobacco (*Nicotiana benthamiana*) was grown in soil in a growth chamber with 16/8-h light/dark photocycle at 24 ± 1°C.

### Measurement of Vine Growth, Berry Growth, and Quality

About 30 shoots from six vines (three from each) were selected for both treatments at budbreak. New Shoot diameter and shoot length were measured by calipers and ruler at an interval of 3 days respectively from −20 days after anthesis. For each treatment, 30 berries from different positions (up, middle and bottom) of 10 clusters were randomly chosen for single berry weight, total soluble solids (TSS) analysis at 10-day intervals during berry development and ripening. About 10 berries in each replicate were randomly collected and weighed. TSS was measured in the juice obtained by crushing the berries of each replicate. TSS was determined by refractometer (Master-M, Atago, Tokyo, Japan) and expressed as°Brix. Glucose, fructose, and sucrose contents were detected by HPLC according to previously described method with minor modification. The extraction of sugar was made as previously described. The juice was centrifuged at 10,000 r/min for 15 min, the supernatant was diluted with 9 ml water and filtered with 0.22 μm syringe filter and prepared for detection. The chromatographic column was prevail carbohydrates (250 × 4.6 mm, 5 μm), the mobile phase was acetonitrile water (75:25, V/V), the flow rate was 0.8 ml/min, the column temperature was 40°C, and the injection volume was 20 μl. Each sample was injected three times, and the corresponding sugar content was calculated according to the obtained standard curve. The average value was taken and the standard deviation was calculated.

### Anthocyanin Measurement in Grape Skin

The extraction of anthocyanin was performed according to the previously described method with minor modification (Wang et al., 2012; Zhang et al., 2013). The grape skin was ground to powder under liquid nitrogen and approximately 1 g powder was added to 5 ml methanol–1% formic acid, and then the solution was sonicated for 10 min and shaken in the dark at 150 rpm, 25°C for 30 min. The mixture was centrifuged for 10 min at 9,000 *g* at 4°C, the supernatant was collected for further analysis and the extraction procedure was repeated three times, the collected supernatants were pooled and evaporated at 37°C in an evaporator machine. The residual material was resuspended in 5 ml ethanol water containing 2% formic acid and 10% methanol. The anthocyanin content was measured at 530 and 657 nm and calculated using the following equation: (A530–A657)/Fw, where Fw represents the fresh weight of grape berry, and all experiments were performed three replicates.

### RNA Extraction and qRT-PCR

Total RNA was extracted using RNAprep Pure Plant Kit (Polysaccharides and Polyphenolics-rich) (TIANGEN, Beijing) according to manufacturer’s introduction. The purity and integrity were analyzed by Nanodrop2000 (Thermo scientific, Wilmington, DE, United States), 5 μg of total RNA was reverse-transcribed using EasyScript@One-step gDNA Removal and cDNA Synthesis SuperMix (TransGen Biotech, Beijing) to obtain the first strand cDNA. qRT-PCR system with a final volume of 20 μl was consisted of 2 μl cDNA, 10 μl rTaq enzyme, 6 μl ddH_2_O, and 1 μl forward and reverse primers, respectively, and performed on a CFX connect Real Time PCR Detection System (Bio-Rad). *VvActin* and *Fv26S* was selected as internal reference for data normalization. qRT-PCR was performed using the following program: 95°C for 30 s, followed by 40 cycles of 95°C for 15 s, 55°C for 15 s, and 60°C for 15 s. The relative expression level of target genes was calculated using 2-^△△*Ct*^ method (Ma et al., 2014, 2015). The specific primers used for the tested genes were designed using Primer Premier 5.0 and listed in Supplementary Table 1.

### Yeast One Hybrid

Yeast one-hybrid assay was applied to investigate the binding of TFs on the promoter of target genes, yeast one-hybrid assay was conducted as previously described (Li et al., 2010; Zhang et al., 2018), the full-length of VvMYB15 and VvWRKY40 was cloned and inserted into pB42AD vector to construct expression vector, the upstream 2,000 bp of start codon ATG of candidate structural genes were cloned into *pLacZ* vector to construct reporter vector, the primers used for vector construction were listed in Supplementary Table 2. Various combinations of expression vector and reporter vector were co-transformed into the yeast strain EGY48 and grown on SD/-Trp/-Ura medium at 30°C for 3 days. The clones were subsequently grown on SD/-Trp/-Ura medium at 30°C for 1 days to test interactions between DNA and protein.

### Dual-LUC Assay

Dual-LUC assay was applied to investigate the transactivation of TFs on the promoter of target genes. The full-length sequences of VvMYB15 and VvWRKY40 was amplified and inserted into pGreen II 62-SK vector to construct expression vector, respectively. The upstream 2,000 bp of start codon ATG of *VvF3*′*5*′*H* and *VvUFGT* were constructed into pGreen II 0800-LUC vector to construct reporter vector, the primer used for vector construction were listed in Supplementary Table 2. All constructs were transformed into *Agrobacterium tumefaciens* GV3101psoup strain, and the cultures were adjusted to an OD600 of 0.6 with infiltration buffer. To study the activity of TFs on the promoter of target genes, a mixture of *A. tumefaciens* harboring expression vector and reporter vector was infiltrated into *N. benthamiana* leaves by needle-less syringe, the infiltrated tobacco held for 48 h in dark, and then exposed to light for 3 h, before imaging, 8 μl luciferin was applied to the position injected with *A*. *tumefaciens* and adapted in darkness for 2 min. On the one hand, the LUC imaging was visualized using Tanon gel imaging software. On the other hand, disks from the tobacco leaves were collected and the enzyme activity of firefly and renilla luciferase was measured using Dual Luciferase Reporter Gene Assay Kit (Yeasen, China). For every TF-promoter interaction, three biological replicates were performed for individual experiment.

### Prediction and Analysis of Protein Structure

Multiple sequence alignments were generated using ClustalX version 2.0 with default parameters. The evolutionary relationship of VvMYB protein with Arabidopsis thaliana were analyzed by MEGA 7.0. A phylogenetic tree was constructed using neighbor-joining (NJ) and maximum likelihood (ML) methods with bootstrap 1000 using MEGA version 7.0. The upstream 2,000 bp sequences of the *VvF3*′*5*′*H* and VvUFGT transcription start site were downloaded from Ensemble plant^1^ and using plant CARE^2^ to predict *cis-*acting regulatory elements in promoter region.

### Yeast Two-Hybrid

Yeast two-hybrid was applied to investigate the interaction of proteins. The yeast-two-hybrid assay was performed according to the previously described method (Lian et al., 2011; Li et al., 2020). The full-length cDNA sequence of VvMYB15 was amplified and cloned into pLexA to generate bait vector, the full-length of coding sequence of VvWRKY40 was also inserted into pB42AD vector to construct prep vectors, the primers used for vector construction were listed in Supplementary Table 2. Bait vector, prep vector, and the p8op-LacZ reporter vector were co-transformed into yeast strain EGY48 and grown on SD/-Ade-His-Leu-Trp medium at 30°C for 3 days. The clones were subsequently grown on SD/-Ade-His-Leu-Trp medium at 30°C for 1 day to verify the interactions between pairs of proteins.

### Bimolecular Fluorescence Complementation Assay

The BiFC assay was performed according to the previously described (Xu et al., 2019), construct expressing pXY104 and pXY106 fusions were applied in BiFC assay. The full-length coding sequence of VvMYB15 and VvWRKY40 were amplified and inserted into pXY104 and pXY106 vector to generate pXY104-VvMYB15, pXY104-VvWRKY40, and pXY106-VvMYB15, pXY106-VvWRKY40, respectively. All constructs were transformed into *A*. *tumefaciens* GV3101 strain and infiltrated into *N. benthamiana* leaves in the given combination. The infiltrated leaves were incubated at 23°C in darkness for 48 h and then exposed to light for 3 h and subjected to the expression of fluorescence observation under a confocal microscopy (Leica TCS SP5II). The primers used in this assay were listed in Supplementary Table 2.

### Co-immunoprecipitation Assay

The Co-IP assay was conducted according to previously described (Lian et al., 2018; Xu et al., 2019), the cDNA full length of VvMYB15 was cloned and inserted into *PHB*:*Flag* to generate *PHB*:*VvMYB15*-*Flag*, the cDNA full length of VvWRKY40 was amplified and inserted into *PHB*:*YFP* to generate *PHB*:*VvWRKY40*-*YFP*. *A. tumefaciens* GV3101 containing the *PHB*:*VvMYB15*-*Flag* was infiltrated into the tobacco leaves alone or together with *pHB*:*VvWRKY40*-*YFP*. The samples were homogenized in lysis buffer [50 mM Tris–HCl, (pH 7.5), 150 mM NaCl, 0.2% Trition-X-100] containing 1 mM Pefabloc and cocktail, and 50 μM MG132. After centrifugation, the supernatant was incubated for 4 h at 4°C with 20 μl of protein G magnetic beads (bed volume, GE Healthcare), which had previously been incubated at 4°C for overnight with 10 μl of anti-GFP antibody (GeneScript). The immunoprecipitates were washed two times with lysis buffer and eluted by boiling with 2 × SDS loading buffer for 5 min. The eluates were subjected to western blot analysis with anti-Flag (Sigma) and anti-GFP (Abmart) antibodies.

### Transient Overexpression in Strawberry

*Agrobacterium tumefaciens* cultures were prepared using the same method detailed for grape berries. For strawberry fruits, the suspensions expressing *PHB:VvMYB15-GFP* and *PHB:VvWRKY40-GFP* and empty vector *PHB:GFP* (for control) were injected into whole fruit, respectively, the primers used for this assay are the same as transient overexpression in grape berries. The total anthocyanin content and the expression pattern of key structural genes in anthocyanin biosynthesis pathway were analyzed at 5 days after infiltration, anthocyanin measurement were conducted according to previously described (Lian et al., 2018). All experiments were performed in three biological replicates. The expression level of key structural genes in anthocyanin biosynthesis pathway were calculated using 2-^△△*Ct*^ method (Dongmei et al., 2021).

### Transient Overexpression in Grape Berries

The construct of *PHB:GFP* containing VvMYB15 and VvWRKY40 was used for transient overexpression. All the construct were independently transformed into *A. tumefaciens* strain GV3101. Attached fruit of similar size at the green stage were selected and injected with *A. tumefaciens* containing construct *PHB:VvMYB15-GFP*, *PHB:VvWRKY40*-*GFP*, and empty vector *PHB:GFP* under the same infiltration conditions, the cultures were adjusted to an OD600 of 0.6 with infiltration buffer. *A. tumefaciens* suspension was evenly injected into the grape berry until the pedicels became hydrophanous, the grape berries were collected 7 days after injection and each fruit was collected as an individual sample, three biological replicates were conducted to carry further analysis, the primers for transient expression are listed in Supplementary Table 2.

### Statistical Analysis

Data obtained in the study was subjected to analysis of one-way analysis of variance (ANOVA) using SPSS 20.0 software. The results were presented as mean ± standard error of three biological replicates. Asterisk represents significantly differences at *P* ≤ 0.05 level, while two asterisks represent significantly differences at *P* ≤ 0.01 level.

## Results

### Analysis of Physiological Parameter

**FIGURE 1:**
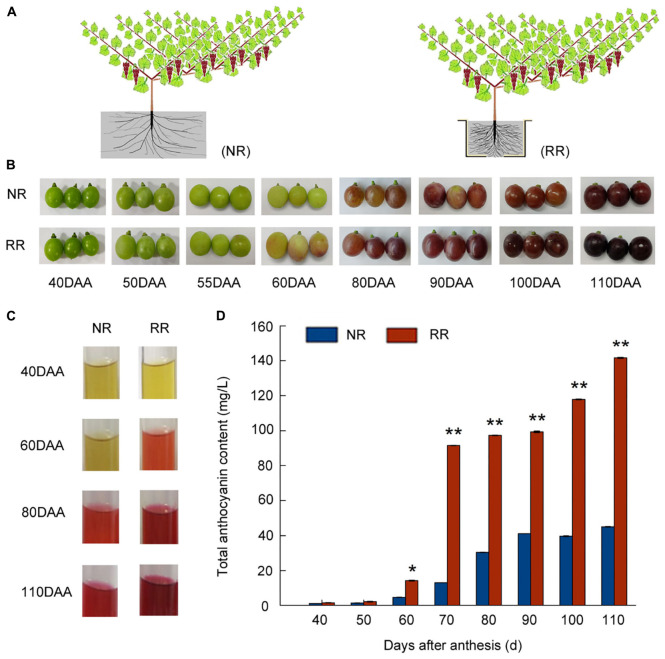
Effects of root restriction cultivation on fruit development and anthocyanin accumulation during grape berry development: **(A)** model of root restriction (RR) and no root restriction cultivation (NR, control); **(B)** process of berry development in grape berries under root restriction cultivation (RR) and no restriction cultivation (NR, control); **(C)** anthocyanin staining; and **(D)** relative anthocyanin contents of grape berries under root restriction (RR) and no root restriction (NR) cultivation. Three biological replicates were performed. Lines graphs and error bars represent average and SE, respectively. Asterisks represent different level of significance (*P* ≤ 0.05*, *P* ≤ 0.01**), and significant differences are indicted by different lowercase letters based on independent sample *t*-test.

The model of root restriction and the control cultivation were depicted in Figure 1A. The vegetative growth of grapevines between root restriction and control throughout sampling stages were observed. New shoot length was measured from 20 to 105 days after anthesis (DAA) (Supplementary Figure 1A) in response to root restriction, the results indicated that the new shoot length under root restriction was significantly lower than that of control and reach about 210 cm. For the base diameter of new shoot (Supplementary Figure 1B), similar trend was observed with the length of new shoot and it was always lower than those of control in response to root restriction at almost whole period and reached at 8.23 cm at maturity, which was consistent with the phenotype of root restriction. Horizontal diameter, vertical diameter of berry, and single berry weight did not show any significant changes in both cultivations at maturity (Supplementary Figures 1C,D,E). The process of grape berries development and ripening in response to root restriction were depicted in Figure 1B and used for further analysis. Meanwhile, we also tested the glucose, fructose and sucrose content in the control and root restriction by HPLC assay, we found that the glucose and fructose content was progressively increased from 55–110 DAA in both root restriction and control, at 110 DAA, glucose and fructose content were significantly higher than those of control, respectively (Supplementary Figures 1F,G). Since no detectable sucrose in both cultivations during all sampling stages, it was not present in the results. The photograph of extraction process of anthocyanin in grape skin is shown in Figure 1C. Regarding to grape berry development, veraison was defined as the onset of ripening in which grape clusters began to color, in this study, almost no detectable anthocyanin in 40 and 50 DAA, and anthocyanin accumulation was initiated at 60 DAA (Veraison), the grape berries were harvested at 110 DAA and total anthocyanin content of grape berry was significantly increased by root restriction, up to 3.2-fold compared with the control (Figure 1D), consistent with our visual appearances, supporting a role for root restriction in promoting anthocyanin accumulation.

### Identification and Expression Patterns of *VvMYB15*, *VvWRKY40*, and Genes Involved in the Anthocyanin Metabolic Pathway

**FIGURE 2:**
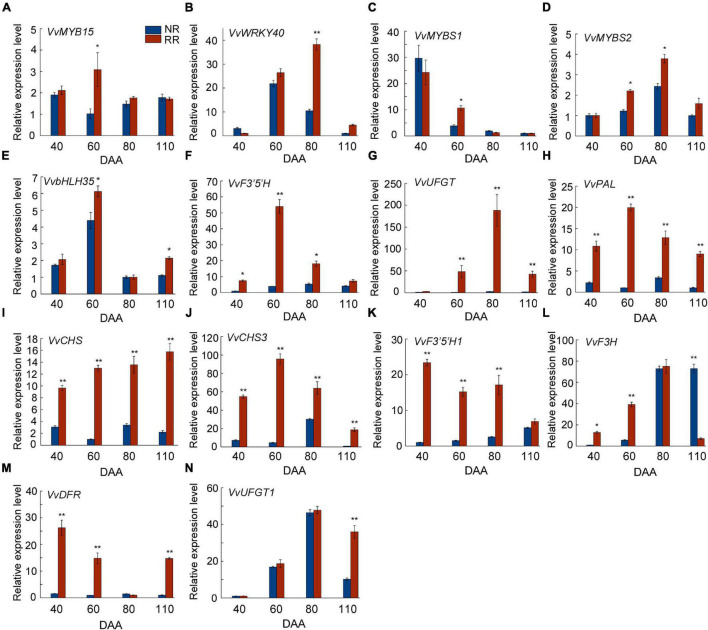
Analysis of expression level of genes related to anthocyanin biosynthesis under root restriction (RR) and no root restriction (NR), **(A)** *VvMYB15*, **(B)** *VvWRKY40*, **(C)** *VvMYBS1*, **(D)** *VvMYBS2*, **(E)** *VvbHLH35*, **(F)** *VvF3*′*5*′*H*, **(G)** *VvUFGT*, **(H)** *VvPAL*, **(I)** *VvCHS*, **(J)** *VvCHS3*, **(K)** *VvF3*′*5*′*H 1*, **(L)** *VvF3H*, **(M)** *VvDFR*, and **(N)** *VvUFGT1*. Asterisks represent different level of significance (*P* ≤ 0.05*, *P* ≤ 0.01**), and significant differences are indicted by different lowercase letters based on independent sample *t*-test.

In our previous transcriptome data, the expression level of *VvMYB15* and *VvWRKY40* and some key structural genes in the anthocyanin biosynthesis pathway were significantly higher in root restriction compared with the control (Leng et al., 2016; Supplementary Figure 2), expression pattern of most these genes were consistent with the change in total anthocyanin content, consequently, we speculated that these differentially expressed genes might be involved in anthocyanin biosynthesis under root restriction. To analyze the possibility of the participation of these differentially expressed genes in anthocyanin accumulation in grape berries under root restriction, expression level of some genes related to the anthocyanin biosynthesis such as *VvMYB15*, *VvWRKY40*, *VvPAL*, *VvCHS*, *VvF3*′*5*′*H*, *VvF3H*, and *VvUFGT* were verified in grape berries during fruit development in response to root restriction (Figure 2). The results showed that the expression pattern of *VvMYB15*, *VvF3*′*5*′*H*, *VvPAL*, and *VvCHS3* were sharply increased at veraison and then gradually decreased until maturity and in parallel with the content of anthocyanin, however, the expression pattern of *VvWRKY40*, *VvUFGT, VvF3H*, and *VvUFGT1* were strongest in post-veraison under root restriction, peaking at 80 DAA. It is a well-known MBW complex, and was involved in anthocyanin biosynthesis in plants, thus, we hypothesized that *VvMYB15* and *VvWRKY40* might be involved in the anthocyanin biosynthesis under root restriction by regulating above structural genes. In addition, we found that those genes were constitutively expressed in different organs, such as bud berries, root, stem, leaf, and flower, especially higher expression was observed in grape berries (Supplementary Figure 3).

### The Transcription Factor VvMYB15 and VvWRKY40 Activates the Promoter of Anthocyanin Biosynthetic Genes

**FIGURE 3:**
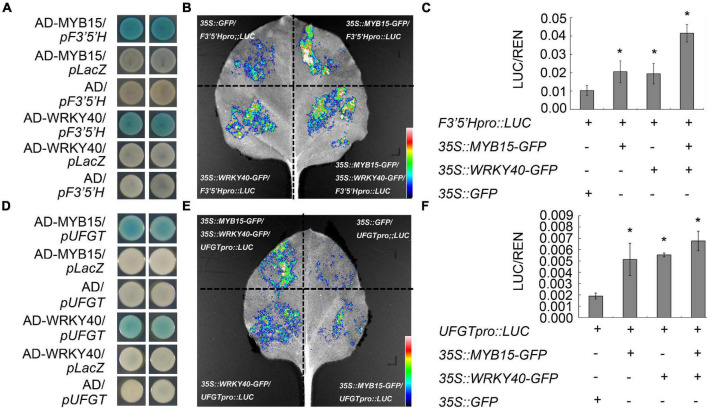
VvMYB15 and VvWRKY40 specifically bind to the promoter region of anthocyanin biosynthesis-related genes. **(A)** Yeast-one-hybrid showing VvMYB15 and VvWRKY40 bind the promoter of *VvF3*′*5*′*H*, respectively. **(B)** Yeast one hybrid showing VvMYB15 and VvWRKY40 bind the promoter of *VvUFGT*, respectively. Blue color represents the expression of *LacZ* reporter gene. Analysis of the relative fluorescence intensity shown in panels **(C,D)**, the fluorescence intensity of control was used as reference. **(E)** VvMYB15 and VvWRKY40 activated the expression of *VvF3*′*5*′*H*. *Nicotiana benthamiana* leaves were co-infiltrated with Agrobacterium strains carrying the indicated constructs. Control (*35S::GFP*/*F3*′*5*′*Hpro::LUC*), co-expression of *35S::VvMYB15-GFP/F3*′*5*′*Hpro::LUC*, co-expression of *35S::VvWRKY40-GFP*/*F3*′*5*′*Hpro::LUC*; co-expression of *35S::VvMYB15-GFP/35S::VvWRKY40-GFP*/*F3*′*5*′*Hpro::LUC*. **(F)** VvMYB15 and VvWRKY40 activated the expression of *VvUFGT*. *Nicotiana benthamiana* leaves were co-infiltrated with Agrobacterium strains carrying the indicated constructs. Control (*35S::GFP*/*UFGTpro::LUC*), co-expression of *35S::VvMYB15-GFP/UFGTpro::LUC*; co-expression of *35S::VvWRKY40-GFP*/*UFGTpro::LUC*; co-expression of *35S::VvMYB15-GFP/35S::VvWRKY40-GFP*/*UFGTpro::LUC*. Expression values were determined by calculating the ratio of LUC activity to REN activity (LUC/REN). The error bars show average ± SE of six biological replicates. “+” indicates the presence of corresponding protein, “–” indicates the absence of corresponding protein. Asterisks represent different level of significance (*P* ≤ 0.05*), and significant differences is indicted by different lowercase letters based on independent sample *t*-test.

To examine if VvMYB15 and VvWRKY40 regulates the expression level of anthocyanin biosynthetic genes, and according to the result of qRT-PCR and total anthocyanin content, *VvPAL* (Phenylalanine ammonia lyase), *VvCHS* (Chalcone synthase), *VvF3*′*5*′*H* (Flavonoid 3′5′-Hydroxylase), *VvUFGT* (Glucosyltransferase), and *VvF3*′*5*′*H 1* (Flavonoid 3′5′-Hydroxylase 1) were selected as candidate genes, therefore, the interaction between VvMYB15, VvWRKY40, and anthocyanin biosynthetic genes were confirmed by a yeast-one-hybrid assay. The promoter of key enzyme genes related to anthocyanin biosynthesis was fused to *pLacZ* vector and VvMYB15 and VvWRKY40 were fused to the *pB42-AD* vector, respectively, when fused *pPAL-LacZ*, *pCHS-LacZ*, *pF3*′*5*′*H-LacZ*, *pUFGT-LacZ*, *pF3*′*5*′*H1-LacZ*, and *pUFGT1-LacZ* were co-expressed with *AD-VvMYB15* or *AD*-*VvWRKY40* in EGY48 yeast strains, we found that yeast containing *AD-VvMYB15* or *AD*-*VvWRKY40* plus *pF3*′*5*′*H-LacZ* or *pUFGT-LacZ* exhibited a much darker blue, in contrast, the color of negative control containing *pB42AD* and *pF3*′*5*′*H*-*LacZ*, *pUFGT*-*LacZ* or *pB42AD-MYB15*, *pB42AD-WRKY40*, and *pLacZ* did not change, which indicated that *VvMYB15* and *VvWRKY40* could directly bind to the promoter of *VvF3*′*5*′*H* and *VvUFGT* (Figures 3A,D), but not the *VvPAL*, *VvCHS3*, *VvF3*′*5*′*H 1*, and *VvUFGT1* (Supplementary Figure 4). To further confirm whether *VvF3*′*5*′*H* and *VvUFGT* genes were activated by VvMYB15 and VvWRKY40, respectively. We used a Dual-LUC system to identify how *VvMYB15* and *VvWRKY40* affects the expression of *VvF3*′*5*′*H* and *VvUFGT*. The coding sequence of MYB15 and VvWRKY40 were amplified and cloned into *pHB:GFP* vector, a 2,000 bp promoter region of *VvF3*′*5*′*H* and *VvUFGT* were fused to upstream of LUC reporter, respectively (Supplementary Figure 5). The effector containing *pHB*:*VvMYB15-GFP* or *pHB:VvWRKY40-GFP* alone or both were co-expressed with *proF3*′*5*′*H:LUC* or *proUFGT:LUC* reporter in tobacco leaves, when *pHB*:*VvMYB15-GFP* or *pHB:VvWRKY40-GFP* alone or both were expressed with *proF3*′*5*′*H:LUC* and *proUFGT:LUC* in tobacco leaves, respectively, the qualitative and quantitative data analyses showed that the expression of reporter *proF3*′*5*′*H:LUC* were significantly higher than negative control (Figures 3B,C), similar result was acquired from reporter *pgroUFGT:LUC* (Figures 3E,F). Collectively, suggesting that VvMYB15 and VvWRKY40 could positively regulate the expression of *VvF3*′*5*′*H* and *VvUFGT*.

### The Ectopic Expression of *VvMYB15* and *VvWRKY40* Promotes Anthocyanin Accumulation in Strawberry

**FIGURE 4:**
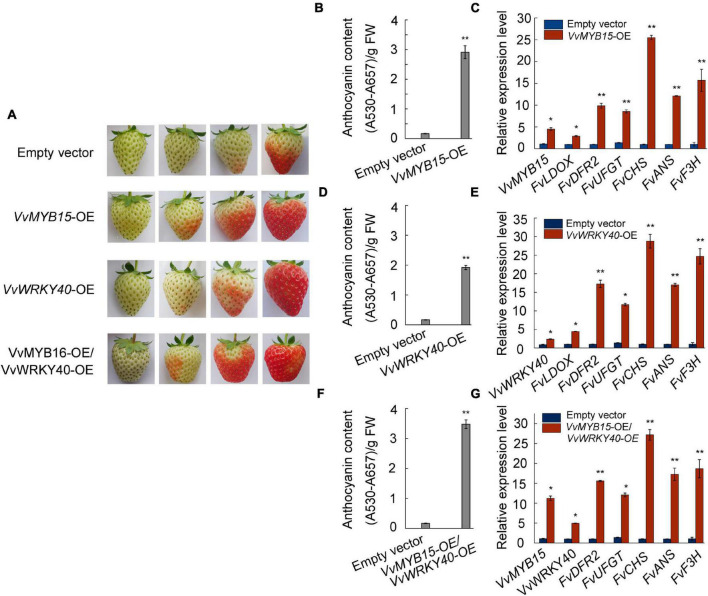
*VvMYB15* and *VvWRKY40* promoted the anthocyanin accumulation in strawberry fruit. **(A)** Anthocyanin accumulation phenotypes and **(B,D,F)** relative anthocyanin contents of strawberry fruit of transient transformed *A. tumefaciens* harboring *35S-GFP* empty (Control), *35S::VvMYB15*-*GFP*, *35S::VvWRKY40*-*GFP* or *35S::VvMYB15*-*GFP*/*35S::VvWRKY40*-*GFP*. The experiments were performed three times with similar results, and a representative picture is shown. **(C,E,G)** Expression analysis of *VvMYB15*, *VvWRKY40* and anthocyanin biosynthesis-related genes (*FvLDOX*, *FvDFR2*, *FvUFGT*, *FvCHS*, *FvANS*, and *FvF3H*) in strawberry fruits were detected by qRT-PCR, *26S* was used as internal control. Three biological replicates were performed, the error bars donate standard deviations. Asterisks represent different level of significance (*P* ≤ 0.05*, *P* ≤ 0.01**), and significant differences is indicted by different lowercase letters based on independent sample *t*-test.

To test whether *VvMYB15* and *VvWRKY40* can promote the anthocyanin accumulation, attached strawberry fruit of similar size at large green period were selected and transiently expressed *VvMYB15* and *VvWRKY40* alone or together by injection of *A. tumefaciens* containing *pHB*:*VvMYB15*-*GFP* and *pHB*:*VvWRKY40*-*GFP* construct. Overexpression of *VvMYB15* significantly promoted anthocyanin accumulation, 3 days after infiltration, *VvMYB15*-OE fruit start to turn red, whereas the control was beginning to turn red after 5 days. On seventh day, *VvMYB15*-OE fruit became fully red, whereas the control fruit reached approximately intermediate red stage, the fruit was harvested 7 days after infiltration (Figure 4A). Further, total anthocyanin content was significantly increased by 13-fold compared to the control (Figure 4B). The expression level of *VvMYB15* was significantly increased in *VvMYB15*-OE fruit, up to 4.5-fold compared to the control, indicating transient overexpression was very effective (Figure 4C). Additionally, the expression level of anthocyanin biosynthesis structural genes including *FvLDOX*, *FvDFR2*, *FvUFGT*, *FvCHS*, *FvANS*, and *FvF3H* were also increased by 3- to 25-fold compared to the control (Figure 4C). Unfortunately, although total anthocyanin content was significantly increased in *WRKY40*-OE fruit (Figure 4D), there was no difference in the expression level of *VvWRKY40* between the negative control and *WRKY40*-OE fruit (Figure 4E). But *VvMYB15* and *VvWRKY40* were co-expressed in strawberry fruits, the expression of anthocyanin biosynthesis structural genes were significantly increased by 5- to 27-fold (Figure 4F) and anthocyanin content was significantly by 25-fold (Figure 4G). The above data supported a role for *VvMYB15* and *VvWRKY40* in promoting anthocyanin accumulation.

### Overexpression of *VvMYB15* and *VvWRKY40* in Grape Berries

**FIGURE 5:**
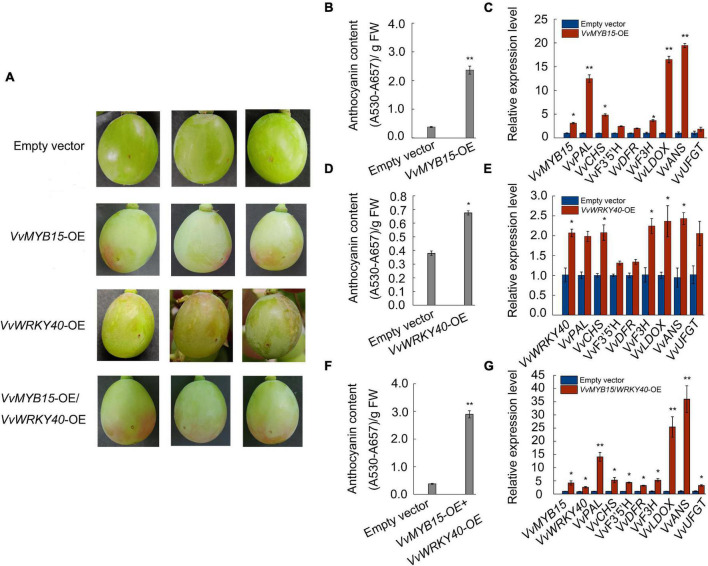
Transient overexpression of *VvMYB15* and *VvWRKY40* promoted anthocyanin accumulation in grape berries, respectively. **(A)** Anthocyanin accumulation phenotypes at 5 days after injection and **(B,D,F)** relative anthocyanin contents of grape berries of transient transformed *A. tumefaciens* harboring *35S::GFP* empty (Control), *35S::VvMYB15*-*GFP*, *35S::VvWRKY40*-*GFP* or *35S::VvMYB15*-*GFP*/*35S::VvWRKY40*-*GFP*, respectively. **(C,E,G)** Expression analysis of *VvMYB15*, *VvWRKY40* and anthocyanin biosynthesis-related genes (*VvPAL*, *VvCHS*, *VvF3*′*5*′*H*, *VvDFR*, *VvF3H*, *VvLDOX*, *VvANS*, and *VvUFGT*) in grape berries were detected by qRT-PCR, *Vvactin* was used as internal control. Three biological replicates were performed, the error bars donate standard deviations. Asterisks represent different level of significance (*P* ≤ 0.05*, *P* ≤ 0.01**), and significant differences is indicted by different lowercase letters based on independent sample *t*-test.

To elucidate the relationship between *VvMYB15*, *VvWRKY40*, and anthocyanin accumulation, we transiently expressed *VvMYB15*, *VvWRKY40* alone or both by injection of *Agrobacterium tumefaciens* containing *VvMYB15*-GFP, *VvWRKY40*-GFP construct driven by 35S promoter into attached green grape berries, overexpression of *VvMYB15*-GFP could significantly increased anthocyanin accumulation, 5 days after infiltration, *VvMYB15*-OE berry start to turn red near the injection site, whereas the negative control was still green (Figure 5A). Further, total anthocyanin content of VvMYB15-OE was up to 6.2-fold higher compared with the control berries (Figure 5B). Expression analysis demonstrated that the empty vector infection did not influence the expression level of *VvMYB15*, while the expression level of *VvMYB15*, *VvPAL, VvCHS, VvF3H, VvLDOX*, and *VvANS* were increased markedly in *VvMYB15-OE* berries and up to 2.5- to 16-fold (Figure 5C). Further, overexpression of *VvWRKY40*-GFP could significantly increase anthocyanin accumulation, up to 1.8-fold compared with the control berries (Figure 5D), supporting a role for *VvWRKY40* in promoting anthocyanin accumulation. Meanwhile, we checked the expression levels of gene encoding enzymes of anthocyanin pathway by qRT-PCR and found *VvWRKY40*, *VvPAL*, *VvCHS*, *VvF3H*, *VvLDOX*, *VvANS*, and *VvUFGT* showed 2-fold higher levels of expression relatively (Figure 5E). Further, total anthocyanin content was significantly increased in VvMYB15/VvWRKY40-OE berries (Figure 5F). An expression analysis demonstrated that the empty vector infection did not influence the expression level of *VvMYB15* and *VvWRKY40*, while the expression level of *VvMYB15*, *VvWRKY40*, *VvPAL*, *VvCHS*, *VvF3*′*5*′*H*, *VvDFR*, *VvF3H, VvLDOX*, and *VvANS* were increased markedly in *VvMYB15/VvWRKY40-OE* berries (Figure 5G), indicating that *VvMYB15* and *VvWRKY40* play an important role in anthocyanin accumulation.

### VvMYB15 Interacts With VvWRKY40 in Yeast Cell and Plant Cell

**FIGURE 6:**
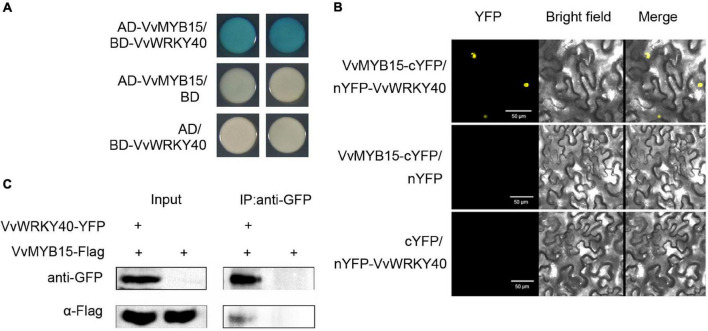
Interaction of VvMYB15 with VvWRKY40 in yeast cell and plant cell. **(A)** Yeast two hybrid showing the interaction between VvMYB15 and VvWRKY40. **(B)** Bimolecular fluorescence complementation assay showing the interaction between VvMYB15 and VvWRKY40 in tobacco cells. Bars = 50 μm. The images show overlays of fluorescence and light views. **(C)** Co-IP assay showing VvMYB15 and VvWRKY40 can interact in tobacco cell. VvMYB15-Flag and VvWRKY40-YFP or VvMYB15-Flag were co-expressed in tobacco leaves.

Since VvMYB15 and VvWRKY40 can directly bind to the promoter of *VvF3*′*5*′*H* and *VvUFGT*, respectively, and previous study showed that MYB, WD40, and bHLH could form complex to regulate the expression of target genes. Therefore, we want to know whether there is direct interaction between VvMYB15 and VvWRKY40. To test this possibility, the yeast-two-hybrid assay *in vitro* was performed, VvMYB15 and VvWRKY40 were fused to the LexA DNA-binding domain (BD) and pB42AD transcriptional activation domain (AD), respectively, the result showed that the yeast cell containing BD-MYB15 plus AD-WRKY40 exhibited a much darker blue, indicating that VvMYB15 could interact with VvWRKY40 (Figure 6A). To further test whether the interaction of VvMYB15 with VvWRKY40 occurred in plant cells, bimolecular fluorescence complementation (BiFC) assay was performed in tobacco leaves, strong YFP signal was observed in tobacco cells when co-expressed VvMYB15-cYFP and nYFP-VvWRKY40, but not in those co-expressing nYFP and MYB15-cYFP or cYFP and nYFP-WRKY40, suggesting a positive interaction was occurred in VvMYB15 and VvWRKY40 (Figure 6B). Furthermore, we performed Co-IP in tobacco cell co-expressing VvMYB15-Flag and VvWRKY40-YFP and found that immunoprecipitation of MYB15-Flag co-immunoprecipitated WRKY40-YFP (Figure 6C), which further demonstrated that VvMYB15 interacts with VvWRKY40 to form heterodimer in plant cells. Thus, on the basis of these results, we suggested that VvMYB15 can form complex with VvWRKY40.

## Discussion

Anthocyanins function as protectants under biotic and abiotic stress in plant life, and contribute to the red-purple coloration for horticultural plant organs, which is also a key parameter in determining external quality. In the past few years, the mechanism underlying anthocyanin biosynthesis have been well unveiled. A complex containing a MYB, bHLH, and WD40 protein is necessary for the appropriate regulation of the anthocyanin biosynthesis in various plant species (Baudry et al., 2004; Gonzalez et al., 2008; Pattanaik et al., 2010). The involvement of MYB TFs in anthocyanin accumulation have been widely verified. However, AtMYB15, as a SG2-type R2R3-MYB transcription factor, was reported to be involved in defense-induced lignification and basal immunity in Arabidopsis (Chezem et al., 2017). In chili pepper, silencing of the R2R3-MYB transcription factor CaMYB resulted in most structural genes including *CHS*, *CHI*, *F3H*, *F3*′*5*′*H*, *DFR*, *ANS*, and *GST* repression and the loss of anthocyanin accumulation (Zhang et al., 2015). VvMYB15 was found to be transcriptional regulation of stilbene biosynthesis in grapevine (Ciaffi et al., 2019). SlMYB15 has been found to be involved in the direct regulation of HY5 on CBF pathway and ultimate cold tolerance in tomato (Zhang et al., 2020). And MYB15 has been found to be involved in regulating plant responses to environmental stresses, such as drought, salt, and cold, the lack of knowledge associated with the molecular regulatory mechanisms of VvMYB15 on anthocyanin accumulation in response to abiotic stress still needs further investigation. And WD40 gene, an important component of MBW, has been rarely studied in grapevine. The PhAN11 gene in petunia is the first WD40 gene isolated in plants, which affects petunia flower color by regulating the transcription of anthocyanin biosynthesis gene (deVetten et al., 1997). Earlier work in apple revealed MdWRKY11 could promote the accumulation of flavonoids and anthocyanins in the apple callus (Wang et al., 2018), and the promotion of MdWRKY11 on the anthocyanins accumulation in apples is achieved by inducing *MdMYB10*, *MdMYB11* (*Liu et al., 2019*). VvWRKY26 in grapevine physically interacts with MYB5 to form MBW complex affecting the pigment accumulation (Amato et al., 2017, 2019). However, whether VvMYB15 and VvWRKY40 could form MBW complex to regulate anthocyanin accumulation are still unclear in grape berries.

Root restriction is considered to be an important cultivation techniques, when the crops were cultivated in root restriction, which was in the condition of stress and stress release, previous report indicated that anthocyanin accumulation was significantly increased in horticultural crops such as grape (Wang et al., 2013a,b; Leng et al., 2018), peach (Pang et al., 2018), apple (Atkinson et al., 2000), and sweet cherry (White et al., 2001) under root restriction, but most of them were only in the physiological stage. In our recent study, it was indicated that anthocyanin content of grape berry was significantly increased under root restriction (Figures 1C,D). Further, the data from RNA-Seq (Leng et al., 2020; Supplementary Figure 2) and qRT-PCR (Figure 2) indicated that the expression profile of some transcription factors including *VvMYB15*, *VvWRKY40* as well as anthocyanin biosynthetic genes such as *VvPAL*, *VvCHS*, *VvF3*′*5*′*H*, and *VvUFGT* were significantly increased under root restriction compared to the control and positively concomitant with the anthocyanin content in grape berries, thus, we speculated that VvMYB15 and VvWRKY40 may be involved in the anthocyanin biosynthesis by regulating these structural genes in grape berry under root restriction. Transcription factors generally regulate the anthocyanin accumulation by directly binding to the promoter of anthocyanin biosynthetic genes. In our study, we also demonstrated that VvMYB15 and VvWRKY40 could directly bind to the promoters of *VvF3*′*5*′*H* and *VvUFGT* by yeast one hybrid assays, respectively (Figures 3A,D), however, VvMYB15 and VvWRKY40 could not directly bind to the promoters of other early anthocyanin biosynthetic genes, such as *VvPAL* and *VvCHS* (Supplementary Figure 4), while data from the dual-luciferase assays performed in this study suggested that the transcriptional activity of *VvF3*′*5*′*H* and *VvUFGT* promoter is directly up-regulated by VvMYB15 and VvWRKY40, respectively (Figures 3B,C,E,F), indicating the activation of *VvPAL* and *VvCHS* are not directly driven by VvMYB15 and VvWRKY40 and other TFs may be involved. Meanwhile, we also carried out *cis-*elements analysis of *VvF3*′*5*′*H* and *VvUFGT* and found that MYB-binding sites and W-box motif were presented in the upstream 2,000 bp, which could explain why VvMYB15 and VvWRKY40 could bind to the promoters of *VvF3*′*5*′*H* and *VvUFGT* and further influence the anthocyanin accumulation. A large number of previous studies have shown that MYB, bHLH, and WD40 can form complex to regulate anthocyanin biosynthesis (Hichri et al., 2011). For instance, In Arabidopsis, TTG1 interacts with both TT2 and TT8 (Antonio Gonzalez et al., 2016), however, in many other species, the WD40 protein only interacts with the bHLH protein and not with the MYB protein (Grotewold et al., 2000; Dubos et al., 2008). In this study, we want to know whether VvMYB15 could form heterodimer with VvWRKY40 to regulate anthocyanin biosynthesis, Y2H (Figure 6A), BiFC (Figure 6B), and Co-IP (Figure 6C) assays proved that VvMYB15 could interact with VvWRKY40, and dual luciferase transient expression assays also showed that co-expression of VvMYB15 with VvWRKY40 significantly induces the expression of *VvF3*′*5*′*H* and *VvUFGT* (Figures 3B,C,E,F), which further confirmed that heterodimer formed by VvMYB15 and VvWRKY40 could significantly activate the expression of *VvF3*′*5*′*H* and *VvUFGT*.

To investigate whether VvMYB15 and VvWRKY40 could regulate anthocyanin accumulation, VvMYB15 and VvWRKY40 was transiently expressed in grape berries and strawberry fruits, overexpression of VvMYB15 and VvWRKY40 alone or together in “Muscat Hamburg” grape berries could significantly promote the anthocyanin accumulation and increased the expression level of anthocyanin biosynthetic genes (Figure 5), however, interference of VvMYB15 and VvWRKY40 did not change anthocyanin accumulation and expression level of anthocyanin biosynthetic genes (the data is not presented), it is likely that PFGC5941 vector cannot be used to silence genes in “Miguang” (*Vitis* vinifera × *Vitis labrusca*) and “Muscat Hamburg” (*Vitis vinifera* L.) grape berries or the system has no function in grape berries. And the similar result was observed in *VvMYB15*-OE “Hongyan” strawberry fruits. However, a complete difference was occurred in VvWRKY40-OE between grape berries and strawberry fruits, it is probably caused by species diversity. In previous study, Gao Zhen found that TRV vector has no function in “Kyoho” grapes (Gao et al., 2018), which further indicated that transient overexpression may be associated with species and variety.

Some external or internal factors affecting *F3*′*5*′*H* and *UFGT* expression have also been identified. It was pointed out that the transcription of *F3*′*5*′*H* and *UFGT* were significantly induced by ABA treatment (Sun et al., 2019), in our previous research, the transcript level of *VvF3*′*5*′*H* and *VvUFGT* was stimulated by root restriction (Wang et al., 2015), ABA content was significantly induced by root restriction in various tissues in grapevine (Guo et al., 2014; Leng et al., 2018). Here, ABRE element was identified in the promoter region of *VvF3*′*5*′*H* in this study, indicating that the expression of *VvF3*′*5*′*H* might be regulated by both ABA and transcription factor. Further studies will be necessary to clarify the relationship between ABA and this regulatory pathway in response to root restriction.

## Conclusion

**FIGURE 7:**
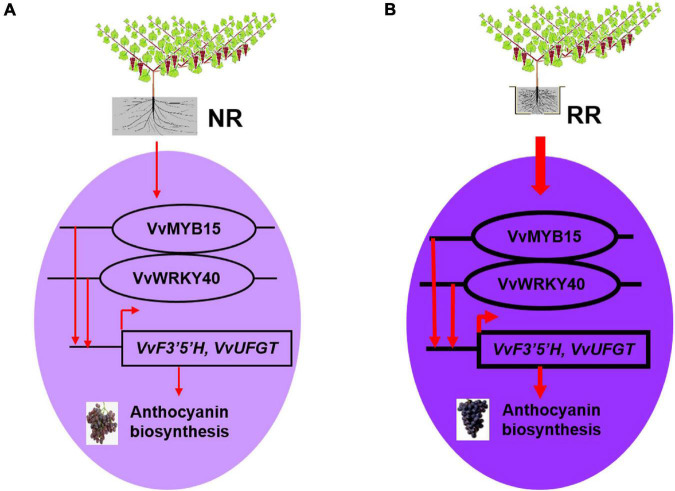
A model of VvMYB15 and VvWRKY40-modulated biosynthesis of anthocyanin under root restriction in grape berries. **(A)** In the absence of root restriction, the expression profile of *VvMYB15* and *VvWRKY40* in grape berries were relatively lower, therefore, the regulatory effect on the downstream anthocyanin biosynthetic genes (*VvF3*′*5*′*H*, *VvUFGT*) was relatively lower, the anthocyanin content of grape berries was relatively lower. **(B)** In the presence of root restriction, the expression level of *VvMYB15* and *VvWRKY40* were significantly increased, at the same time, VvMYB15 and VvWRKY40 could form heterodimer that binds the promoter of *VvF3*′*5*′*H* and *VvUFGT* and activate their expression, which contributed to the accumulation of anthocyanin in grape berry. The thicker the arrow, the higher level of the gene expression; the thinner the arrow, the lower level of the gene expression. Purple indicates the higher anthocyanin content, whereas light purple indicates the lower anthocyanin content.

In summary, the data presented here demonstrated that root restriction induced the higher expression of *VvMYB15* and *VvWRKY40*, while VvMYB15 interacts with proteins VvWRKY40, and bind to the promoter regions of anthocyanin biosynthesis genes *VvF3*′*5*′*H* and *VvUFGT* and further activate their expression level, respectively, which in turn induces the anthocyanin accumulation and grape berries coloration (Figure 7).

## Data Availability Statement

The original contributions presented in the study are included in the article/Supplementary Material, further inquiries can be directed to the corresponding author/s.

## Author Contributions

DL and HL conceived the project and conducted on critical reading and manuscript editing. DL and SW prepared the samples. DL and ZW performed the experiments. SS, MC, and KX assisted with data collection and data analysis. DL wrote the manuscript. DL, LW, CM, and CZ assisted with data analysis, critical reading, and manuscript editing. All authors contributed to the article and approved the submitted version.

## Conflict of Interest

The authors declare that the research was conducted in the absence of any commercial or financial relationships that could be construed as a potential conflict of interest.

## Publisher’s Note

All claims expressed in this article are solely those of the authors and do not necessarily represent those of their affiliated organizations, or those of the publisher, the editors and the reviewers. Any product that may be evaluated in this article, or claim that may be made by its manufacturer, is not guaranteed or endorsed by the publisher.

## Footnotes

1
http://plants.ensembl.org/index.html

2
http://bioinformatics.psb.ugent.be/webtools/plantcare/html/
