# Corrigendum: VvMYB15 and VvWRKY40 Positively Co-regulated Anthocyanin Biosynthesis in Grape Berries in Response to Root Restriction

**DOI:** 10.3389/fpls.2022.850160

**Published:** 2022-02-22

**Authors:** Dongmei Li, Zhenping Wang, Sijie Sun, Kun Xiao, Minghao Cao, Xiangyi Li, Chao Ma, Caixi Zhang, Lei Wang, Hongli Lian, Shiping Wang

**Affiliations:** ^1^Department of Plant Science, School of Agriculture and Biology, Shanghai Jiao Tong University, Shanghai, China; ^2^School of Agriculture and Biology, Ningxia University, Yinchuan, China; ^3^Institute of Agro-Food Science and Technology, Key Laboratory of Agro-Products Processing Technology of Shandong, Shandong Academy of Agricultural Sciences, Jinan, China

**Keywords:** anthocyanin, VvMYB15, VvWRKY40, root restriction, grape

In the original article, there was a mistake in Figure 1B as published. All experiments were obtained from “Muscat Hamburg” grape berries. However, the phenotype of the grape berries mistakenly showed other grape variety in Figure 1B. This error is corrected in the new version.

**Figure 1:**
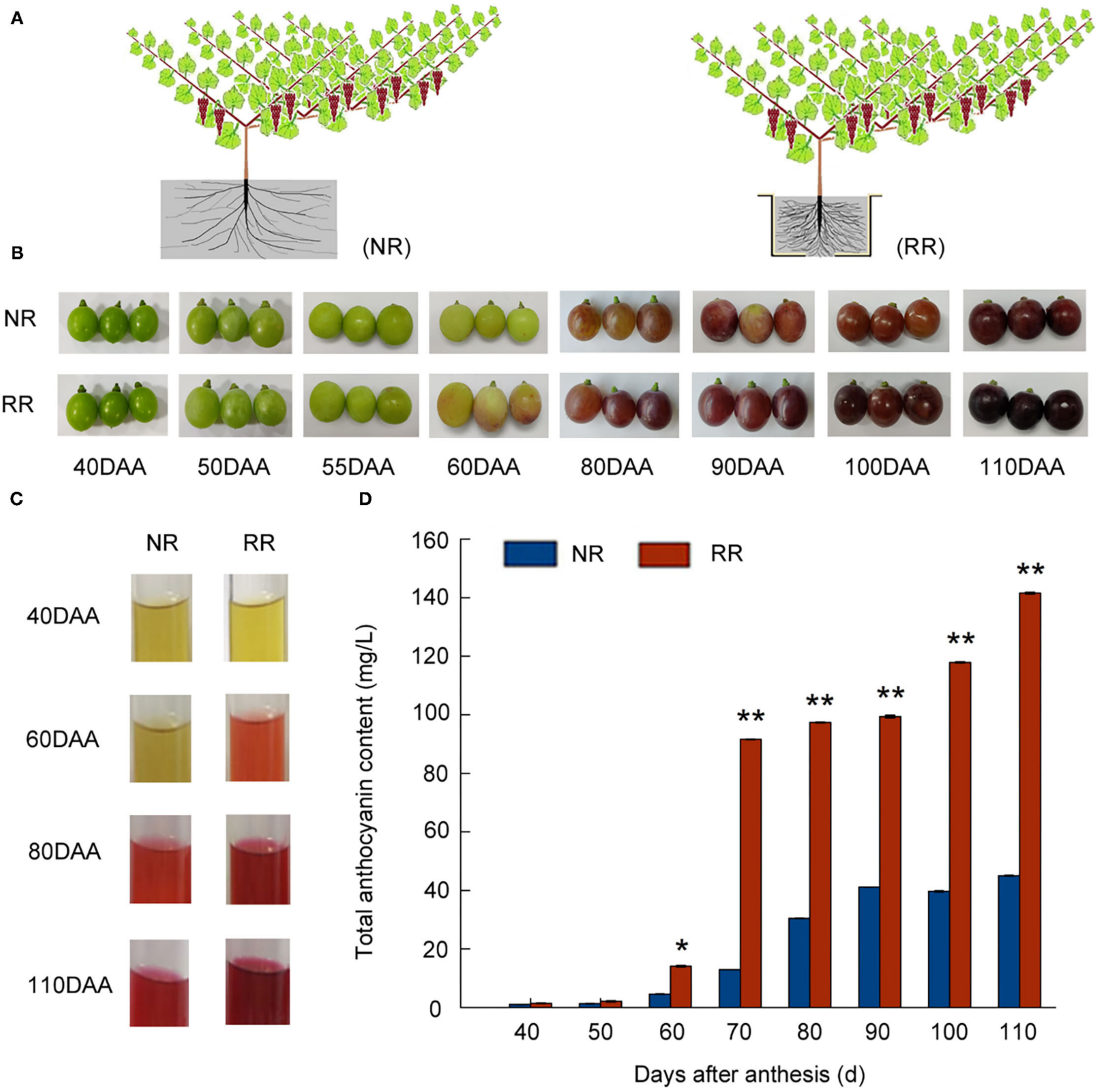
Effects of root restriction cultivation on fruit development and anthocyanin accumulation during grape berry development: **(A)** Model of root restriction (RR) and no root restriction cultivation (NR, control); **(B)** process of berry development in grape berries under root restriction cultivation (RR) and no restriction cultivation (NR, control); **(C)** anthocyanin staining; and **(D)** relative anthocyanin contents of grape berries under root restriction (RR) and no root restriction (NR) cultivation. Three biological replicates were performed. Lines graphs and error bars represent average and SE, respectively. Asterisks represent different level of significance (*P* ≤ 0.05*, *P* ≤ 0.01**), and significant differences are indicted by different lowercase letters based on independent sample *t*-test.

The authors apologize for this error and state that this does not change the scientific conclusions of the article in any way. The original article has been updated.

## Publisher's Note

All claims expressed in this article are solely those of the authors and do not necessarily represent those of their affiliated organizations, or those of the publisher, the editors and the reviewers. Any product that may be evaluated in this article, or claim that may be made by its manufacturer, is not guaranteed or endorsed by the publisher.

