# Supplementary material for: VvMYB15 and VvWRKY40 Positively Co-regulated Anthocyanin Biosynthesis in Grape Berries in Response to Root Restriction
Source: Front Plant Sci. 2021 Dec 9;12:789002. doi: 10.3389/fpls.2021.789002 (PMC8695491; doi:10.3389/fpls.2021.789002)
Supplement: Supplementary file 1 [file Data_Sheet_1.docx]

**Supporting informations**


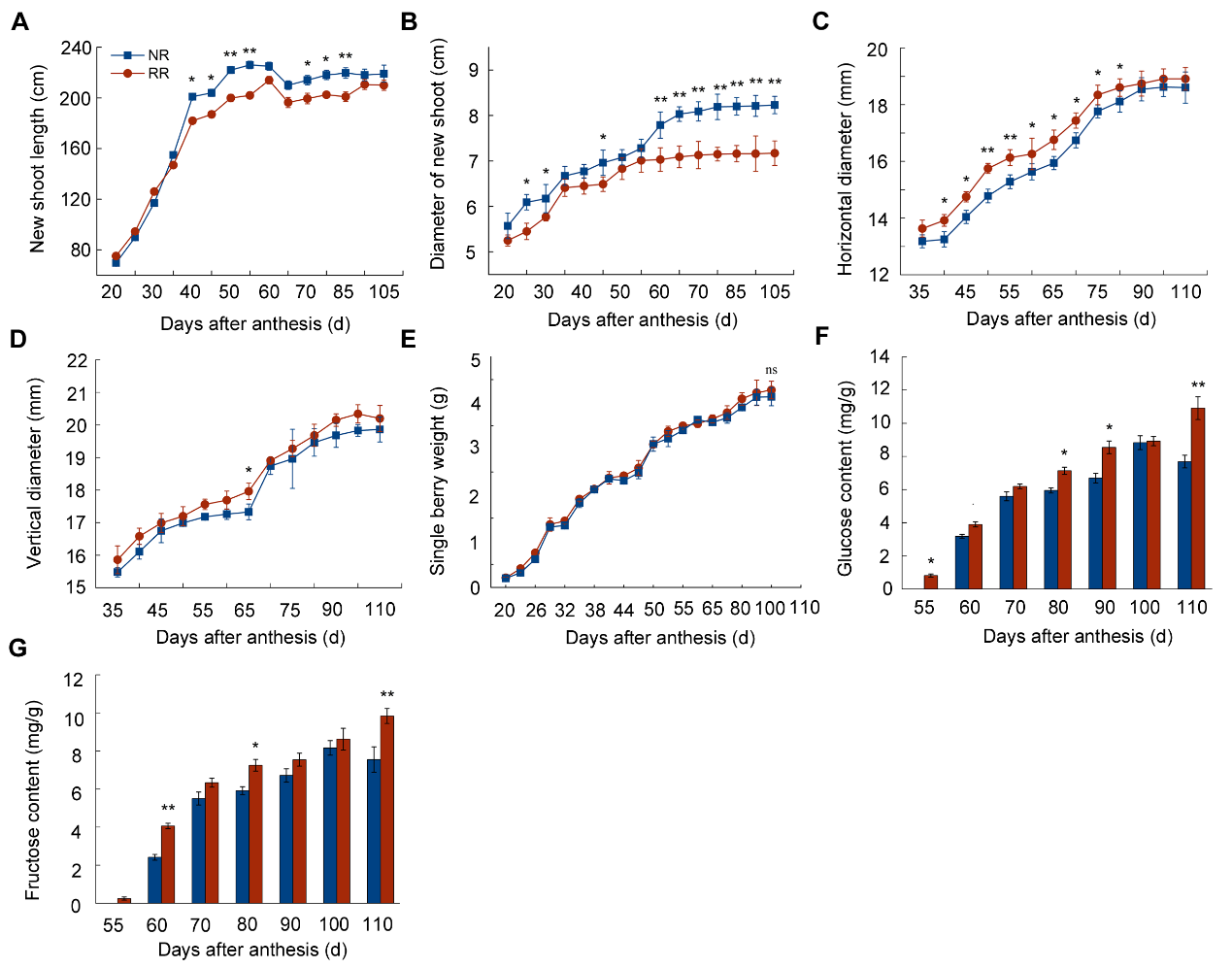


Figure S1. Effect of root restriction on physiological index of grapevine. (A) New shoot length; (B) Diameter of new shoot; (C) Horizontal diameter; (D) Vertical diameter; (E) Single berry weight; (F) Glucose content; (G) Fructose content.


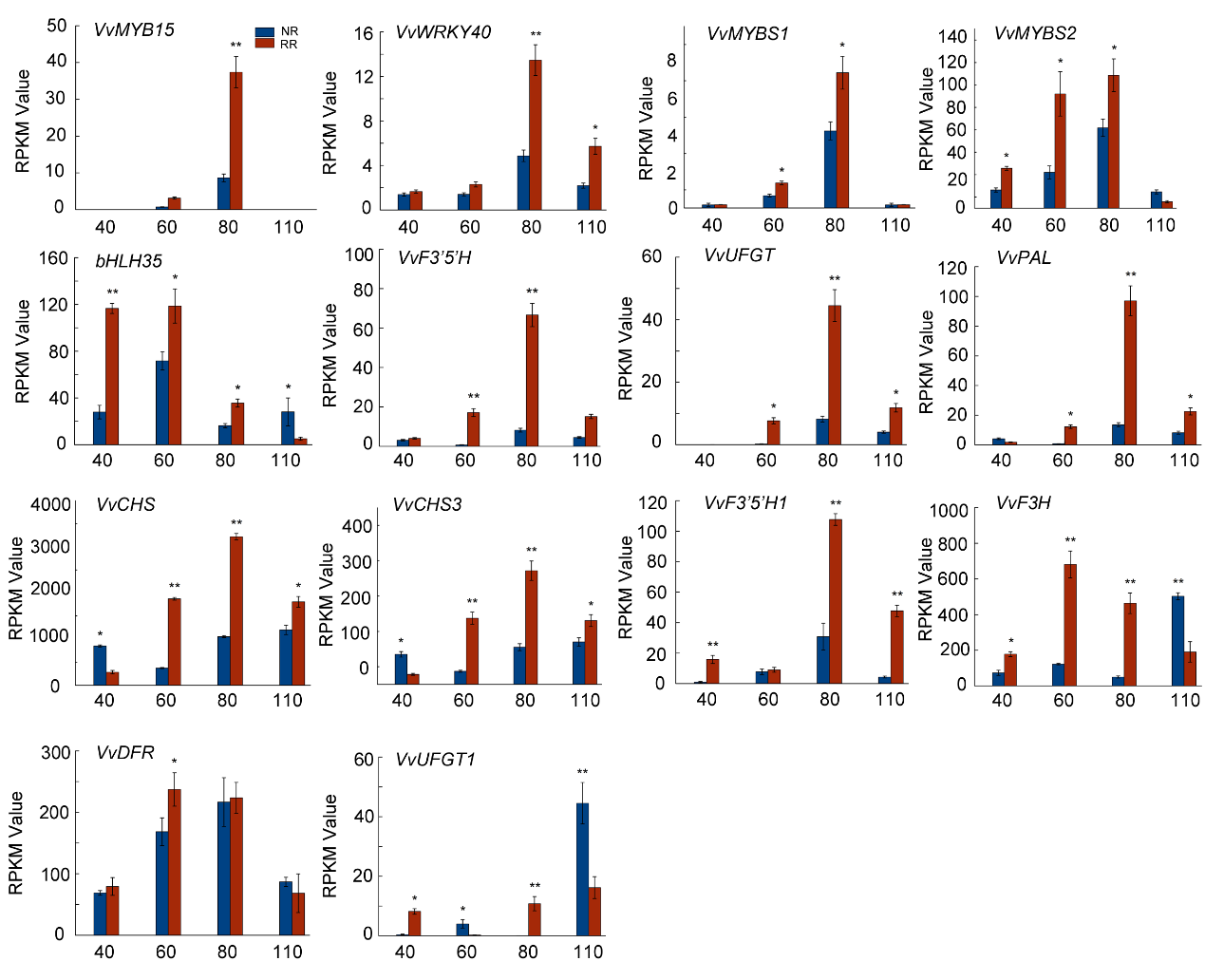


Figure S2. RPKM value of genes related to anthocyanin biosynthesis identified from transcriptome data, *VvMYB15*, *VvWRKY40*, *VvMYBS1*, *VvMYBS2*, *VvbHLH35*, *VvF3’5’H*, *VvUFGT*, *VvPAL*, *VvCHS*, *VvCHS3*, *VvF3’5’H1*, *VvF3H*, *VvDFR*, *VvUFGT1*


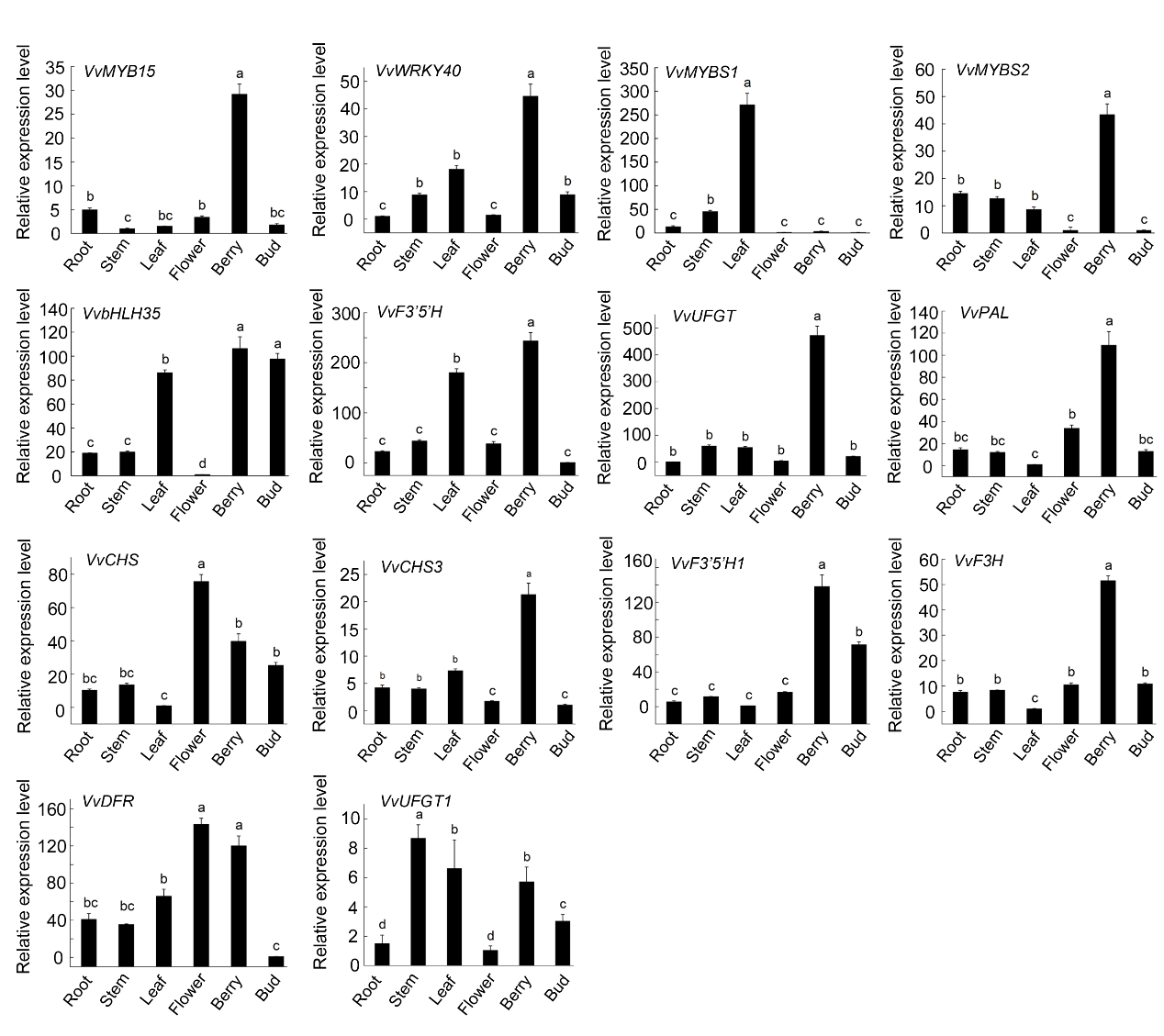


Figure S3. Analysis of tissue-specific expression pattern of genes related to anthocyanin biosynthesis. *VvMYB15*, *VvWRKY40*, *VvMYBS1*, *VvMYBS2*, *VvbHLH35*, *VvF3’5’H*, *VvUFGT*, *VvPAL*, *VvCHS*, *VvCHS3*, *VvF3’5’H1*, *VvF3H*, *VvDFR*, *VvUFGT1*.


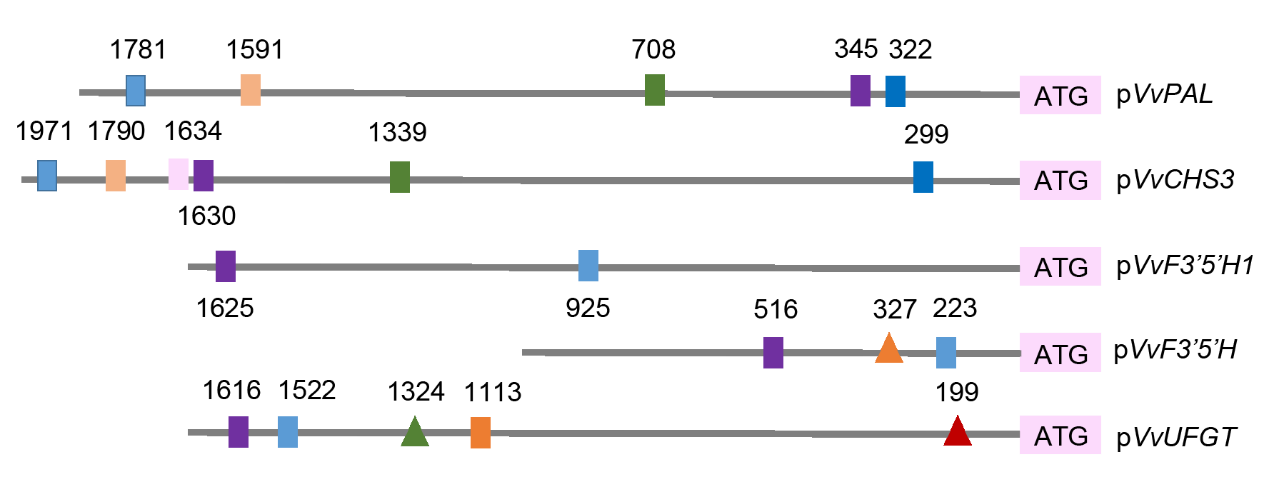


Figure S4. Schematic diagrams of the *VvPAL*, *VvCHS3*, *VvF3’5’H1*, *VvF3’5’H* and *VvUFGT* promoters. The plantCARE database was used to predict cis-acting elements in the 2000bp *VvPAL*, *VvCHS3*, *VvF3’5’H1*, *VvF3’5’H* and *VvUFGT* promoter regions. The rectangle represents MYB binding site and the triangle represents WRKY binding site (W-box).


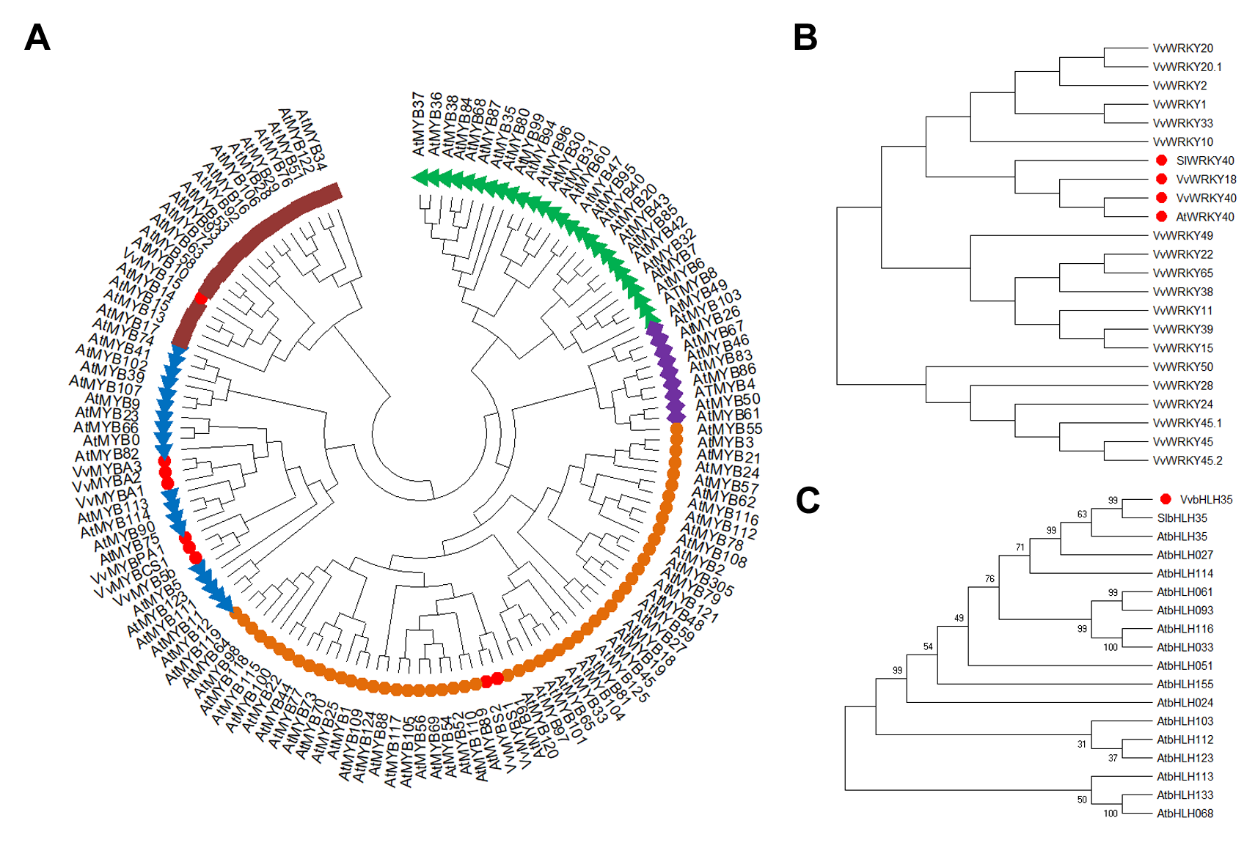


Figure S5. Phylogenetic analysis of MYB, WRKY and bHLH proteins, respectively. The phylogenetic tree was constructed using neighbor-joining (NJ) with bootstrap 1000 using MEGA version 7.0. (A) Phylogenetic analysis of MYB proteins between Arabidopsis thaliana and grape. (B) Phylogenetic analysis of WRKY proteins among Arabidopsis thaliana, tomato and grape. (C) Phylogenetic analysis of bHLH proteins among Arabidopsis thaliana, tomato and grape.


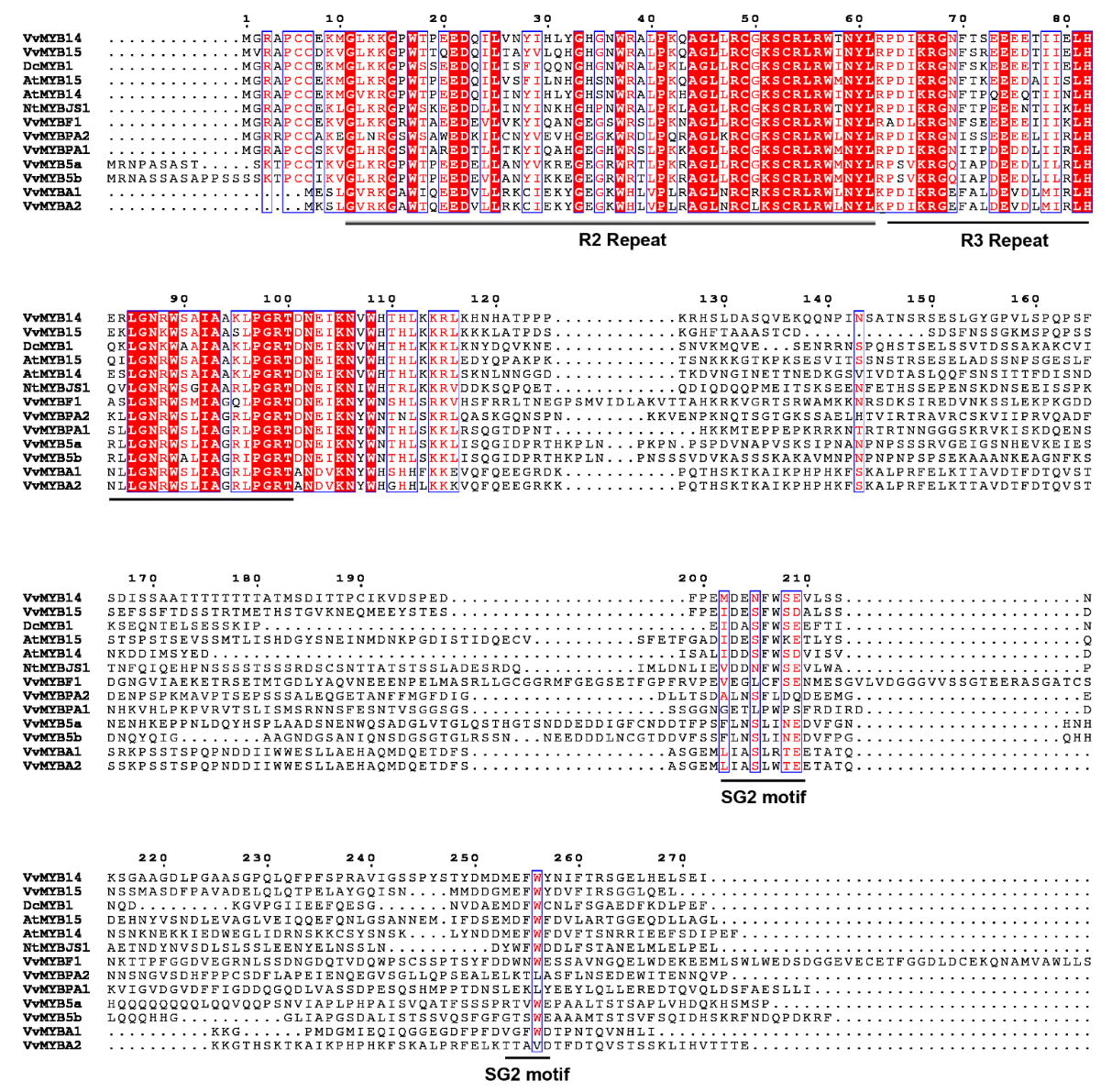


Figure S6. Sequence similarities and conserved domain of VvMYB15 was aligned using Espript 3.0 (<http://espript.ibcp.fr/ESPript/ESPript/index.php>).


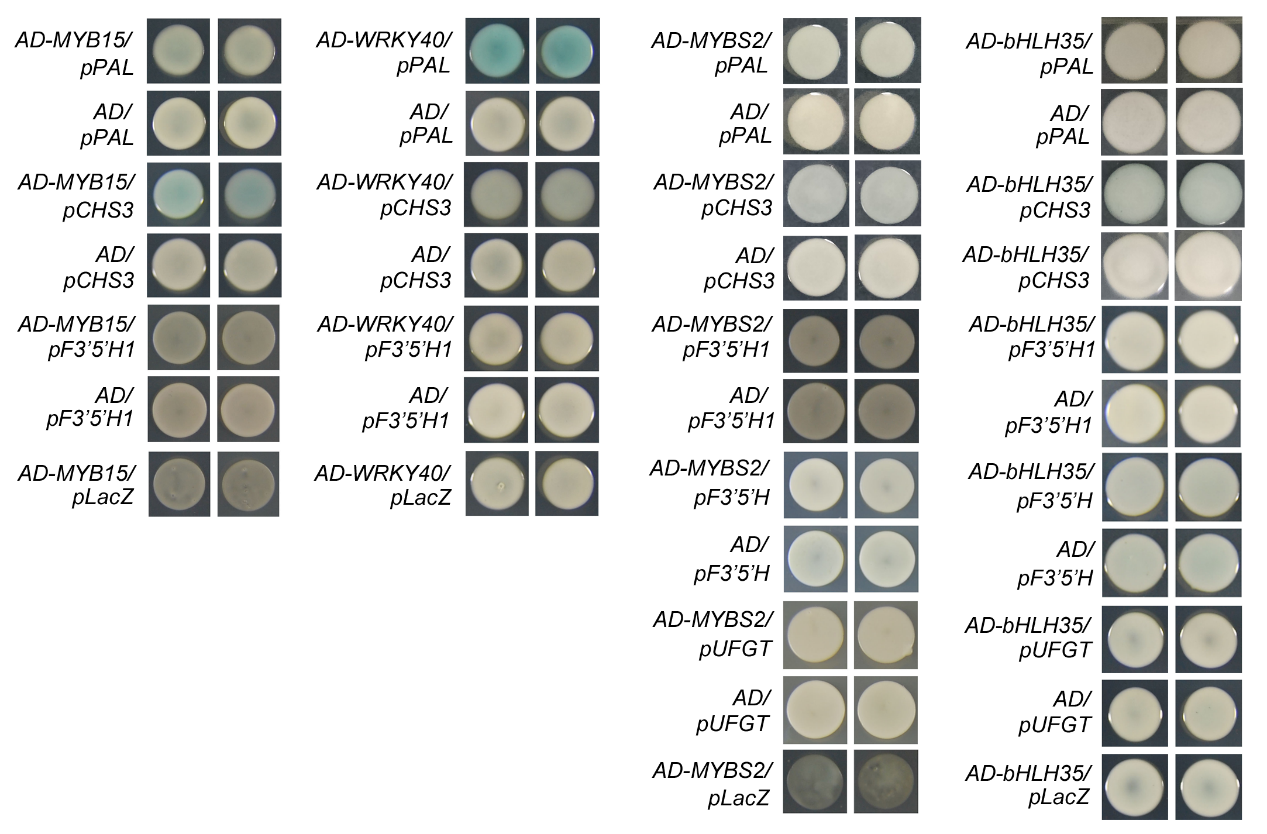


Figure S7. VvMYB15, VvWRKY40 VvMYBS2 and VvbHLH35 bind to the promoter region of anthocyanin biosynthetic genes. VvMYB15 bind the promoter of *VvCHS3*, but not *VvPAL* and *VvF3’5’H1*. VvWRKY40 could bind the promoter of *VvPAL*, but not *CHS3* and *VvF3’5’H1*. VvMYBS2 and VvbHLH35 could not bind the promoter of *VvPAL*, *VvCHS3*, *VvF3’5’H1*, *VvF3’5’H1* and *VvUFGT*, respectively.


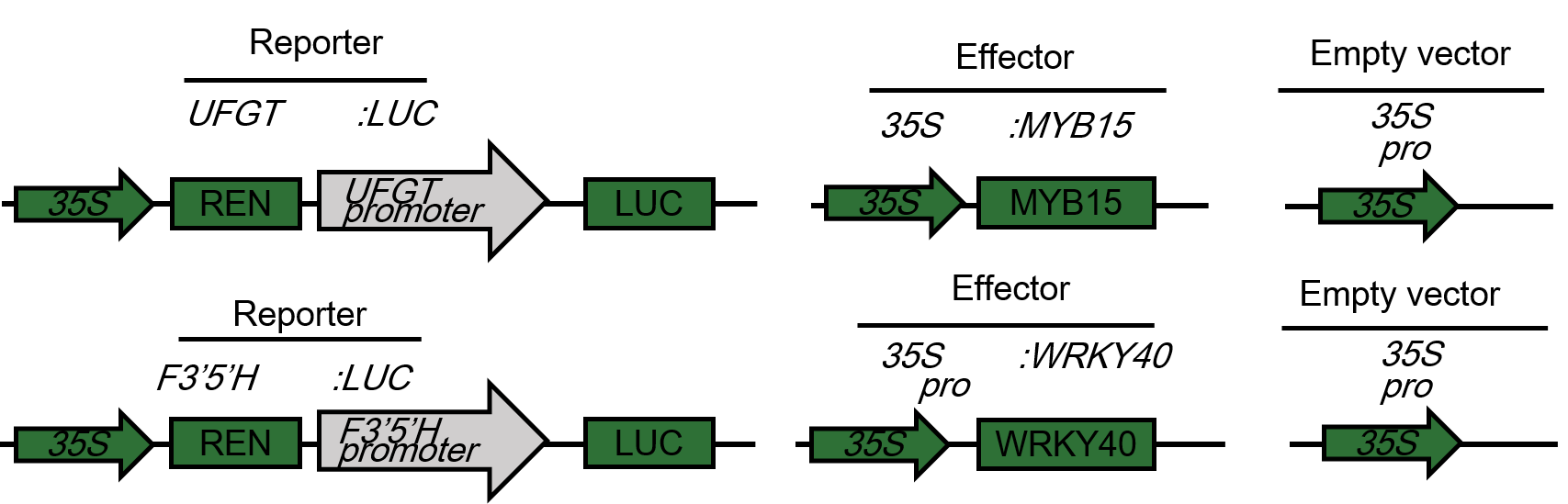


Figure S8. Model of vector construction. The VvF3’5’H and VvUFGT reporter vector was depicted in the left, The VvMYB15 and VvWRKY40 reporter vector was depicted in the right.
