# Supplementary material for: VvMYB15 and VvWRKY40 Positively Co-regulated Anthocyanin Biosynthesis in Grape Berries in Response to Root Restriction
Source: Front Plant Sci. 2021 Dec 9;12:789002. doi: 10.3389/fpls.2021.789002 (PMC8695491; doi:10.3389/fpls.2021.789002)
Supplement: Supplementary file 2 [file Data_Sheet_2.docx]

Table S1. Primers used for reverse-transcription quantitative PCR analysis

|  | Primer-F | Primer-R |
| --- | --- | --- |
| *VvMYB15* | AGTTGCAGACTCCGATGGAT | GGTGTGTGTGCCAGACATTT |
| *VvWRKY40* | TGTGTCAAGGTCCGGATTGA | AATTCGACCAGGGTGTGGAT |
| *VvDFR* | CCGCAAAGACTGTCAGAAGG | CTCAGCCAGTGTCTTGGAGA |
| *VvPAL* | CAACAATGGGTTGCCCTCAA | GCGCTCTGAACATGATTGGT |
| *VvCHS* | CTCTGACACCCACCTGGATT | TGCAGAGACGAGTTCGAAGA |
| *VvCHS3* | TGGAGGTACCAAAGCTAGGC | GCAGCTTGGTGAGTTGGTAG |
| *VvF3’5’H* | GATTTGCGCTGGCACTAGAA | ATGGCCGAAAGAGAAACTGC |
| *VvF3’5’H1* | GATTTGCGCTGGCACTAGAA | ATGGCCGAAAGAGAAACTGC |
| *VvF3H* | GCTTCATCGTTTCCAGCCAT | GAGCTTCTCGCTGTACTCCT |
| *VvUFGT* | AGCACAAGGCCACATCAATC | ACCTCAATCCATCCACGGTT |
| *VvUFGT1* | CATTAGCTTTGGCACCGTCA | CGTACCCTCTGGTCTTCTCC |
| *FvCHS* | GGCTCACCGTCGAGACCG | GGTGAACCCAGATACCTTC |
| *FvDFR* | CACGATTCACGACATTGCGAAATT | GAACTCAAACCCCATCTCTTTCAGCTT |
| *FvUFGT* | CTAAGCAAAGGAAAGTTGAACGGAAT | TCCAACCGCAATGTGTTACAAA |
| *FvLDOX* | GAAGTGCGTACCCAACTCCATCGT | ACCTTCTCCTTGTTGACGAGCCC |
| *FvPAL* | ATGGCGCTTAACGGAAACG | CTAAGATATGGGCAGGGGGGC |

Table S2. Primers used for vector construction. The restriction sites are marked with red font.

|  | Primer sequence |
| --- | --- |
| *45-MYB15-F* | gattatgcctctcccgaattcatggtaagagctccttgttgtgataagg |
| *45-MYB15-R* | agaagtccaaagcttctcgagtcaaagctcctgtaagccgc |
| *45-WRKY40-F* | gattatgcctctcccgaattcatggctatggatagttctaattggatggc |
| *45-WRKY40-R* | agaagtccaaagcttctcgagtcaccatttttcagtttgattatggtggagaat |
| *pLacZ-F3'5'H-F* | tttgatattggatcggaattcagcatggtggtggcctc |
| *pLacZ-F3'5'H-R* | atacagagcacatgcctcgagcatctttgttaataaccagtcgaacttcctatgt |
| *pLacZ-UFGT-F* | tttgatattggatcggaattctaaacaaggtgagacgagatggctat |
| *pLacZ-UFGT-R* | atacagagcacatgcctcgagggttggaatgggggatgttacaatttg |
| *pLacZ-PAL-F* | tttgatattggatcggaattcttattttgttttattttttgttctccaatgggtgtga |
| *pLacZ-PAL-R* | atacagagcacatgcctcgagttgatgatgagtggctgtgtttgg |
| *pLacZ-CHS-F* | tttgatattggatcggaattctaaaaccaaatataagaaaattattttctttaatattttttaagaacc |
| *pLacZ-CHS-R* | atacagagcacatgcctcgagttttggctgcttgaatcagtgtgaac |
| *62SK-MYB15-F* | cgctctagaactagtggatccatggtaagagctccttgttgtgataagg |
| *62SK-MYB15-R* | gtcgacggtatcgataagctttcaaagctcctgtaagccgc |
| *62SK-WRKY40-F* | cgctctagaactagtggatccatggctatggatagttctaattggatggc |
| *62SK-WRKY40-R* | gtcgacggtatcgataagctttcaccatttttcagtttgattatggtggagaat |
| *0800-F3'5'H-F* | gtcgacggtatcgataagcttagcatggtggtggcctc |
| *0800-F3'5'H-R* | cgctctagaactagtggatcccatctttgttaataaccagtcgaacttcctatgt |
| *0800-UFGT-F* | gtcgacggtatcgataagctttaaacaaggtgagacgagatggctat |
| *0800-UFGT-R* | cgctctagaactagtggatccggttggaatgggggatgttacaatttg |
| *202-MYB15-F* | aacggcgactggctggaattcatggtaagagctccttgttgtgataagg |
| *202-MYB15-R* | ttggctgcaggtcgactcgagtcaaagctcctgtaagccgc |
| *202-WRKY40-F* | aacggcgactggctggaattcatggctatggatagttctaattggatggc |
| *202-WRKY40-R* | ttggctgcaggtcgactcgagtcaccatttttcagtttgattatggtggagaat |
| *104-MYB15-F* | attacaggtacccggggatccatggtaagagctccttgttgtgataagg |
| *104-MYB15-R* | cacgctgccaccgccgtcgacaagctcctgtaagccgcc |
| *104-WRKY40-F* | attacaggtacccggggatccatggctatggatagttctaattggatggc |
| *104-WRKY40-R* | cacgctgccaccgccgtcgacccatttttcagtttgattatggtggagaattct |
| *106-MYB15-F* | atcgaggacgccggcggatccatggtaagagctccttgttgtgataagg |
| *106-MYB15-R* | acgaaagctctgcaggtcgactcaaagctcctgtaagccgc |
| *106-WRKY40-F* | atcgaggacgccggcggatccatggctatggatagttctaattggatggc |
| *106-WRKY40-R* | acgaaagctctgcaggtcgactcaccatttttcagtttgattatggtggagaat |
| *pHB-MYB15-Flag-F* | tctctctctaagcttggatccatggtaagagctccttgttgtgataagg |
| *pHB-MYB15-Flag-R* | gctcaccatactagtggatcctcaaagctcctgtaagccgc |
| *pHB-WRKY40-YFP-F* | tctctctctaagcttggatccatggctatggatagttctaattggatggc |
| *pHB-WRKY40-YFP-R* | gctcaccatactagtggatccccatttttcagtttgattatggtggagaattct |
| *pHB-MYB15-GFP-F* | ctccaagcttggatccatggtaagagctccttgttgtgataagg |
| *pHB-MYB15-GFP-R* | tgctcaccatactagtaagctcctgtaagccgcc |
| *pHB-WRKY40-GFP-F* | ctccaagcttggatccatggctatggatagttctaattggatggc |
| *pHB-WRKY40-GFP-R* | tgctcaccatactagtccatttttcagtttgattatggtggagaattct |
| *PFGC5941-MYB15-F* | tctagaggcgcgcctgttgtgataaggtgggattgaagaagg |
| *PFGC5941-MYB15-R* | ggatccatttaaatgttgaggagtctgtaaatgaagagaattcac |
| *PFGC5941-WRKY40-F* | tctagaccatggatggctatggatagttctaattggatggc |
| *PFGC5941-WRKY40-R* | ggatccatttaaatccatttttcagtttgattatggtggagaattct |
